# Relationship, partner factors and stigma are associated with safer conception information, motivation, and behavioral skills among women living with HIV in Botswana

**DOI:** 10.1186/s12889-021-12268-5

**Published:** 2021-12-08

**Authors:** Sarah A. Gutin, Gary W. Harper, Neo Moshashane, Kehumile Ramontshonyana, Rob Stephenson, Starley B. Shade, Jane Harries, Okeoma Mmeje, Doreen Ramogola-Masire, Chelsea Morroni

**Affiliations:** 1 Center for AIDS Prevention Studies, Division of Prevention Science, University of California, San Francisco, 550 16th Street, 3rd Floor, 94143 San Francisco, CA USA; 2 Department of Health Behavior and Health Education, School of Public Health, University of Michigan, 1415 Washington Heights, MI 48109 Ann Arbor, USA; 3 Women’s Health Research Unit, School of Public Health and Family Medicine, Faculty of Health Sciences, University of Cape Town, 7925 Observatory, Cape Town, South Africa; 4 Botswana Harvard AIDS Institute Partnership Princess Marina Hospital, Private Bag BO 320, Gaborone, Botswana; 5 Department of Systems, Population and Leadership, School of Nursing, University of Michigan, MI Ann Arbor, USA; 6 The Center for Sexuality and Health Disparities, School of Nursing, University of Michigan, Ann Arbor, MI USA; 7 Department of Epidemiology & Biostatistics School of Medicine, University of California, San Francisco, CA San Francisco, USA; 8 Department of Obstetrics and Gynecology, University of Michigan Medical School, MI Ann Arbor, USA; 9 Department of Obstetrics and Gynecology, Faculty of Medicine, University of Botswana, Gaborone, Botswana; 10 MRC Centre for Reproductive Health, University of Edinburgh, 47 Little France Crescent, EH16 4TJ Edinburgh, United Kingdom

**Keywords:** Safer conception, Botswana, Information-motivation-behavioral skills, HIV stigma

## Abstract

**Background:**

A significant proportion (20-59%) of people living with HIV in sub-Saharan Africa desire childbearing, are of reproductive age, and are in sero-different relationships (~50%). Thus it is plausible that some portion of new HIV transmissions are due to attempts to become pregnant. Safer conception (SC) methods that effectively reduce the risk of HIV transmission exist and can be made available in resource-constrained settings. Few studies in the region, and none in Botswana, have quantitatively examined the correlates of information, motivation, and behavioral skills for SC uptake.

**Methods:**

We surveyed 356 women living with HIV from 6/2018 to 12/2018 at six public-sector health clinics in Gaborone, Botswana. Participants were 18-40 years old, not pregnant, and desired future children or were unsure about their childbearing plans. We examined correlates of SC information, motivation, and behavioral skills using nested linear regression models, adjusting for socio-demographic, interpersonal, and structural variables.

**Results:**

Knowledge of SC methods varied widely. While some SC methods were well known (medical male circumcision by 83%, antiretroviral therapy for viral suppression by 64%), most other methods were known by less than 40% of participants. Our final models reveal that stigma as well as relationship and partner factors affect SC information, motivation, and behavioral skills. Both internalized childbearing stigma (ß=-0.50, 95%CI:-0.17, -0.02) and perceived community childbearing stigma were negatively associated with SC information (ß=-0.09, 95%CI:-0.80, -0.21). Anticipated (ß=-0.06, 95%CI:-0.12, -0.003) and internalized stigma (ß=-0.27, 95%CI:-0.44; -0.10) were associated with decreased SC motivation, while perceived community childbearing stigma was associated with increased SC motivation (ß=0.07, 95%CI:0.02, 0.11). Finally, internalized childbearing stigma was associated with decreased SC behavioral skills (ß=-0.80, 95%CI: -1.12, -0.47) while SC information (ß=0.24, 95%CI:0.12, 0.36), motivation (ß=0.36, 95%CI:0.15, 0.58), and perceived partner willingness to use SC (ß=0.47, 95%CI:0.36, 0.57) were positively associated with behavioral skills

**Conclusions:**

Low SC method-specific information levels are concerning since almost half (47%) of the study participants reported they were in sero-different relationships and desired more children. Findings highlight the importance of addressing HIV stigma and partner dynamics in interventions to improve SC information, motivation, and behavioral skills.

**Supplementary Information:**

The online version contains supplementary material available at 10.1186/s12889-021-12268-5.

## Background

Botswana has one of the highest burdens of HIV in the world with an estimated prevalence of 18.2 - 22.1% among adults [1]. Between 22.2 and 27.3% of reproductive aged women (15-49 years) are living with HIV [1]. Studies in Botswana have found that while up to 70% of women know they are living with HIV before becoming pregnant [2, 3], 43 - 50% of all women report their pregnancies were unintended [2, 4, 5]. While Botswana has exhibited strong political support for treatment of HIV and has made significant progress towards UNAIDS 95-95-95 targets (namely, that by 2030, 95% of people living with HIV (PLHIV) will know their HIV status, 95% will be on antiretroviral treatment (ART), and 95% will be virally suppressed), the country has struggled to control new HIV infections [6]. Botswana saw a 4% increase in new HIV infections from 2010 to 2017, and it is plausible that some of these new infections are among reproductive-aged sero-different couples who desire childbearing [7]. This increase in new infections highlights the need to promote safer conception (SC) approaches among HIV-affected couples who would like to become pregnant.

Methods that effectively reduce the risk of HIV transmission can be made available in resource-constrained settings to safely achieve pregnancy. Approaches such as timed condomless intercourse and vaginal insemination are conception specific while male circumcision, viral suppression using ART, and pre-exposure prophylaxis (PrEP) are not conception specific methods but effectively reduce the risk of HIV transmission to uninfected partners [8–16]. Studies in various sub-Saharan African countries have found that these methods are acceptable and have been used by HIV-affected couples who desire childbearing [17–22]. However, despite these various options, the use of SC methods by PLHIV who desire pregnancy remains low in sub-Saharan Africa [18, 19, 23].

Although SC options can make achieving pregnancy safer, research suggests that both men and women living with HIV (WLHIV) in sub-Saharan Africa generally have low information about SC strategies, thus limiting their uptake [18, 19, 23–26]. However, studies have shown varying levels of awareness and information about specific SC methods. A recent study in Kenya found an awareness among PLHIV of ART-based methods, sperm washing, self-insemination, and timed condomless intercourse [27]. However, they found limited understanding about how to track one’s fertile days as well as a lack of specific information about sperm washing and self-insemination [27]. Studies in South Africa have reported awareness of sperm washing but little or no awareness of PrEP, self insemination, or timed condomless intercourse as SC options [28, 29]. This suggests that SC information varies by context, making country-specific data imperative for intervention development. To date, we are not aware of published quantitative studies that have reported on knowledge of SC in Botswana, which could guide the provision of SC services.

The use of SC methods may be particularly affected by partner and relationship dynamics. These might include reproductive autonomy (decision-making power, freedom from coercion, and communication within relationships), experiences of intimate partner violence (IPV), and perceptions of a partner’s willingness to use SC methods. Data from various sub-Saharan African countries has highlighted the influence of partners on fertility desires and SC method utilization, with male partners acting as both a facilitator and barrier to greater uptake [18, 24, 25, 30–34]. While male involvement in SC has been low in existing SC programs [18], some studies have found that men report a willingness to attend clinics with female partners for SC services [35]. Having support from one’s intimate partner to use a SC method is critical, and the perceived willingness of partners to use SC methods has been linked to greater motivation to use SC [25]. Identifying partner-related factors that can be targeted in interventions would likely help to improve SC method uptake.

Reproductive decisions among PLHIV often take place in a context of extensive societal stigma [36–39]. Sub-Saharan African studies have found that PLHIV often face anticipated, perceived, and/or experienced stigma from healthcare providers when pregnant or trying to have children [37, 40–43] and this can inhibit women’s communication about fertility desires [44–48]. Our prior qualitative research in Botswana found that internalized stigma and anticipated stigma from providers hinder women from seeking SC services [34, 38]. In addition, despite strong family and community expectations to have children in many sub-Saharan African contexts [30, 49–52], studies have reported strongly perceived community disapproval associated with HIV and reproduction, and community pressure on WLHIV to not have children [30, 53, 54]. Quantitatively establishing the types of stigma that WLHIV in Botswana experience, and the effect of that stigma, can help identify possible intervention targets.

As noted above, there may be various reasons why WLHIV do not seek SC strategies and services, including deficits or barriers to information, motivation, or behavioral skills for SC. A theoretical approach that is guided by the Information, Motivation, and Behavioral skills (IMB) model can inform the understanding of how factors such as context and culture may affect IMB. The IMB model is a meditational model that conceptualizes the psychological determinants of HIV preventive behavior and provides a framework for understanding preventive behaviors across populations [55, 56]. The model has been utilized for various HIV-preventive behaviors, including engagement in HIV care services [57] and in high HIV prevalence settings internationally [58–61]. In the context of SC, IMB can help us understand how information (e.g., knowledge of SC methods generally and specifically [18, 25]), motivation to use SC (e.g., personal motivation which may be driven by fertility desires, partner willingness to use SC, unequal power dynamics in relationships, and community-level and healthcare provider stigma regarding childbearing [25, 30, 41, 62–64]), and behavioral skills (e.g., self-efficacy, pregnancy planning skills, optimizing health before attempting conception, and specific skills related to SC method use [32, 36, 42, 61, 65]) can lead to SC method utilization [55, 56].

To develop SC interventions that meet the needs of HIV-affected couples, it is necessary to understand the correlates of SC information, motivation, and behavioral skills to identify areas and types of clients that can be targeted to promote SC. Few studies in the region, and none in Botswana, have quantitatively examined the correlates of information, motivation, and behavioral skills for SC uptake [25]. To address this gap, we assessed the factors that influence these SC constructs among WLHIV in Botswana.

## Methods

### Study Design and Setting

A quantitative, cross-sectional survey was administered to a sample of women aged 18-40 years between June and December 2018 in the greater Gaborone, Botswana area. The survey was administered at six public sector health clinics, including one hospital-based referral clinic, three clinics in low-income urban areas, and two clinics in middle-income peri-urban areas. Sites were chosen in consultation with the Gaborone District Health Management Team (DHMT) and selected because each had a high client volume, offered sexual and reproductive health services, and had a clinic where clients were accessing HIV care and treatment services. Each site provides HIV testing, ART, and family planning and contraceptive services.

### Participants

Women were eligible for the study if they (1) self-reported living with HIV, (2) were between 18 and 40 years old, (3) reported a desire to have children at any time in the future or were unsure about their childbearing plans, and (4) were not currently pregnant. The participant age range of 18-40 was selected because 18 is the legal age of consent in Botswana, and we felt SC would be less relevant for women over age 40 who would likely have lower fertility desires. Partner HIV status and partner disclosure were not eligibility criteria. Although SC methods are especially relevant for sero-different couples, sero-concordant couples who are both living with HIV can still benefit from SC through reduced risks for transmission of resistant virus.

Recruitment procedures used a multi-modal approach. The first approach was to have local research assistants announce and then briefly explain the study to groups of clients in clinic waiting rooms. Interested clients were instructed to approach study staff members so they could be assessed for eligibility. The second approach was that potential participants were informed about the study by health center staff and, if interested, were referred to a study research assistant. In all cases, research assistants screened women for study eligibility and if they met the criteria, the research assistant explained the aims of the study. If the woman agreed to participate, the research assistant read her the consent form in either Setswana or English (based on the preference of the participant) and also gave her time to read the consent form to ensure she understood what was being asked of her. Before beginning the questionnaire, all participants provided written informed consent. Participants were offered a snack and received money per Botswana ethics requirements (30 BWP, approximately 3 USD at the time of the study) to cover local transport costs.

Ethical approvals were obtained from the University of Michigan Health Sciences and Behavioral Sciences Institutional Review Board in Ann Arbor, Michigan (HUM00128900), the University of Botswana Research Ethics Committee, Office of Research and Development in Gaborone, Botswana (HPDME 13/18/1 × 1), the Health Research and Development Division of the Botswana Ministry of Health in Gaborone, Botswana (HPDME 13/18/1), and the Research and Ethics committee of the Princess Marina Hospital in Gaborone, Botswana (PMH 5/79(423-4-2018)). Permission was also obtained from the coordinator of the Gaborone DHMT and the heads of health facilities before recruitment of WLHIV took place. This study complied with all the principles of the Declaration of Helsinki and all methods were performed in accordance with relevant guidelines and regulations.

### Procedures

Trained and experienced local research assistants administered paper-based questionnaires via face-to-face interviews conducted in a private room at the clinic where participants were recruited. All local research assistants were fluent in English and Setswana. Research assistants read all items on the survey aloud to ensure data consistency and account for varying literacy levels. Surveys took approximately 40 min to administer. All paper questionnaires were entered into a custom-designed REDCap data entry system. All data were reviewed after initial entry to check for errors and completeness. After data entry, paper questionnaires were stored in a locked file cabinet in Gaborone at the Botswana-UPenn Partnership offices (University of Botswana campus).

### Measures

The survey was pre-tested (n = 10) in Botswana with women who met the study eligibility criteria, but were not included in the participant sample, to assess understanding and acceptability. Measures were adapted when necessary to suit the local context and to aid comprehension. All measures were administered in either Setswana or English, depending on the preference of the participant. Information about all scales is presented, including where the scale was developed, number of items, response options, scale values, example questions, and adaptations, as appropriate. For all scales assessing underlying constructs using Likert-type items, internal consistency reliability is reported with Cronbach’s alpha scores. All scales mentioned below that are from Uganda were developed for use with sero-concordant and sero-different couples affected by HIV who had intentions to have a child with their partner within the next two years. This is similar to women in the current study who were in either sero-concordant or sero-different relationships and desired a child in the future.

#### Socio-demographic, reproductive, and relationship factors

Socio-demographic, reproductive, and relationship factors included recruitment location, age, educational attainment, main source of income, relationship status (e.g., married, cohabitating, in a relationship but not cohabiting, etc.), and total number of living biological children.

#### SC method information

SC information was measured with an adapted version of a SC method awareness instrument (17-items) developed in Uganda [25]. This instrument assesses awareness of the availability of SC methods in general, conception-specific SC methods (such as sperm washing, manual self-insemination, and timed condomless intercourse) and SC risk reduction strategies that are not conception-specific (circumcision, PrEP, ART adherence, etc.). Respondents were asked to select “True”, “False”, or “Don’t know” in response to a series of statements. An example statement was: “Only having unprotected sex during the few days each month when the woman is most fertile will help to limit the risk of HIV transmission to an uninfected partner”. The sum of correct responses was tabulated to create a total score with higher scores representing higher levels of SC information. We added two additional statements to the original instrument: one about ART adherence for viral suppression and one about medical male circumcision. Although WLHIV would not be able to use SC methods that are meant for sero-different partnerships in which the male partner is living with HIV (such as sperm washing), we wanted to assess their knowledge of the full range of SC options. This measure was administered before asking other SC-related questions to minimize responses being influenced by exposure to other measures.

#### Motivation to use SC methods

SC method motivation was measured using an adapted version of a SC method motivation scale that was developed in Uganda (4-items, original Cronbach’s alpha = 0.88, current sample alpha = 0.88) [25, 61]. This scale assesses a respondents’ level of commitment and readiness to engage in SC counseling or use a SC method. The SC method motivation scale asked questions about desire to make pregnancies safer and was not dependent on knowing about specific SC methods. Item choices were on a 4-point scale ranging from 1 (strongly disagree) to 4 (strongly agree). An example item was: “I am ready to temporarily delay getting pregnant if it helps me have a child more safely”. Item scores were summed to create a total score with higher scores representing higher levels of motivation to use SC methods. Items choices were reduced from a 10-point scale to a 4-point scale. One question was added to the original scale and assessed confidence in receiving quality health care services.

#### Behavioral skills to use SC

The IMB model stipulates that behavioral skills are composed of an individual’s objective ability or skills and perceived self-efficacy concerning performance of the behavior. SC skills were measured using a series of six questions that we developed to assess whether WLHIV felt they had used certain behavioral skills that might aid them in using SC methods (response options were yes/no). An example question is: “Have you ever had a discussion with a healthcare provider about how to make conception safer if you want to become pregnant in the future?” Following each skill question, WLHIV were asked how certain they were that they could engage in the skill that had just been described (6 item scale, Cronbach’s alpha = 0.86). Response options were on a 4-point scale from 1 (I cannot do this at all) to 4 (I can definitely do this) (see Additional file 1 for full scale). An example question was: “How certain are you that you and your current partner could talk about ways to make conception safer if you ever wanted to become pregnant in the future?” Items for this scale were developed based on prior literature and our own qualitative research in Botswana which found that communication skills with providers [35, 47, 66] and partners [34, 67, 68] were particularly salient behavioral skills for SC. Content validity was assessed by submitting items to content experts (two experts in the field of sexual and reproductive health (SRH)/SC (one doctor from Botswana, one researcher from the USA) and two local Botswana researchers with many years of experience in SRH/HIV research in Botswana) for review during survey development. Face validity was explored during pilot testing with volunteers who met the study eligibility criteria. Both sets of questions were combined into scales with higher values denoting (1) higher self-assessment of having the behavioral skills to engage in SC use and (2) higher self-efficacy to engage in the SC behavioral skills that were mentioned. The 12-item scale showed high internal consistency (Cronbach’s alpha = 0.84).

#### Perceived partner willingness to use SC

The respondent’s perception of their partner’s willingness to use a SC method was assessed using a scale developed in Uganda (three items, original Cronbach’s alpha = 0.85, current sample alpha = 0.92) [25]. Item choices were on a 5-point scale from 1 (no confidence) to 5 (high confidence). An example item was: “Your partner would be open to trying methods to reduce HIV transmission risks during conception”. Item scores were summed to create a total score with higher scores representing higher levels of confidence in partner willingness to use SC.

#### Future fertility desires

Future fertility desires were assessed by asking questions from previous research and Demographic and Health surveys [49, 69–71]. Future fertility desires were assessed by asking participants: “Do you want to become pregnant within the next year?” Response options were on a 4-point Likert scale (Definitely not, Probably not, Probably yes, Definitely yes). Those who responded either “Definitely not” or “Probably not”, were then asked, “Do you want to have children, or more children, at any time in the future?” Response options were yes/no/do not know. Women who responded “no” were considered not to desire future children. These questions were coded to create a dichotomous variable (desire more children in the future/ no desire for more children or not sure). Those who said they wanted to be pregnant within the next year (Probably yes, Definitely yes) and those who said they desired children at some time in the future were categorized as desiring future children.

#### Reproductive Autonomy

An adapted version of the Reproductive Autonomy Scale was used [72] to explore issues around interpersonal gender dynamics. This scale was developed in the USA but has been used in sub-Saharan Africa [73, 74] to assesses whether women have the power to decide about and control issues related to contraceptive use, pregnancy, and childbearing. The adapted scale had 12 items that addressed three domains: decision-making, freedom from coercion, and communication) [72]. The adapted decision-making sub-scale (three items, original Cronbach’s alpha = 0.65, current sample alpha = 0.91) asked women about which partner had the final say in different reproductive situations with the following response categories: my sexual partner (or someone else), both me and my sexual partner (or someone else) equally, or me. Participants were allowed to say someone else since parents, in-laws, or others may have the final say about reproductive decisions. An example question was: “Who has the most say about when you have a baby in your life?” One question from the original decision-making sub-scale about abortion and adoption was dropped because abortion is illegal in Botswana and adoption is rare. The adapted freedom from coercion sub-scale (four items, original Cronbach’s alpha = 0.82, current sample alpha = 0.94) was on a 4-point scale ranging from 1 (strongly agree) to 4 (strongly disagree). An example item was: “My partner has pressured me to become pregnant”. One item from the original freedom from coercion sub-scale was dropped because during pre-testing in Setswana, participants thought that two of the questions were asking about the same thing. The communication sub-scale (five items, original Cronbach’s alpha = 0.74, current sample alpha = 0.94) was on a 4-point scale ranging from 1 (strongly agree) to 4 (strongly disagree). An example item was: “If I was worried about being pregnant or not being pregnant, I could talk to my partner about it”. Item scores were reverse coded as necessary and then summed to create a total score with higher scores indicating higher levels of reproductive autonomy.

#### Experiences of lifetime intimate partner physical violence

 An adapted version of the WHO violence against women instrument [75] was used to measure physical violence experienced at any time in the respondent’s life (four items). The scale was developed for use in low and middle-income countries (including sub-Saharan Africa). Item choices were on a 4-point scale ranging from 1 (Never) to 4 (Many times). An example question was: “How many times has a current or previous partner ever hit you with a fist or with something else that could hurt you?” Item scores were summed to create a total score with higher scores representing higher levels of lifetime intimate partner physical violence. Two items from the original scale which asked about being choked, burnt, or threatened with a gun, knife, or other weapon were removed.

#### Anticipated HIV stigma

An adapted version of the HIV stigma measure [76] that was developed in the USA but has been used in sub-Saharan Africa with PLHIV [77, 78] was used to explore the impact of anticipated HIV stigma (five items, Cronbach’s alpha = 0.89, sample alpha = 0.89). Response options were on a 5-point scale ranging from 1 (very unlikely) to 5 (very likely). An example item was: “Family members will avoid me”. Item scores were summed to create a total score with higher scores indicating greater anticipated stigma. The original scale was shortened from nine to five items. Instead of asking about treatment from community/social workers, we asked about treatment from healthcare workers.

#### Internalized childbearing stigma

Internalized stigma towards childbearing was measured with a scale developed in Uganda (2 items, Cronbach’s alpha = 0.72, sample alpha = 0.81) [25]. Respondents were asked to rate their agreement with two statements (“I feel ashamed for wanting to have a child” and “I feel selfish for wanting to have a child”). Response options were on a 5-point scale ranging from 1 (disagree strongly) to 5 (agree strongly). Item scores were summed to create a total score with higher scores representing higher internalized childbearing stigma.

#### Perceived community stigma

Perceived community stigma toward childbearing among PLHIV was measured with a scale developed in Uganda (three items, Cronbach’s alpha = 0.94, sample alpha = 0.97) [61]. This scale assesses respondents’ perception of community stigma surrounding pregnancy and childbearing for HIV-affected couples. Item choices were on a 5-point scale ranging from 1 (disagree strongly) to 5 (agree strongly). An example item was: “People in the community look down on people living with HIV who want to have a child”. Item scores were summed to create a total score with higher scores representing higher perceived community-level stigma.

### Data Analysis

All data were analyzed using Stata 16 (Stata Corporation, College Station, TX, USA). The amount of missingness in the data was examined and found to be minimal (between 0 and 2.53%). We checked for possible collinearity of variables, and excluded some variables from models because of concerns about multicollinearity (collinear variables included relationship status, HIV disclosure status to partner, total pregnancies, and number of living children). When faced with collinear variables, decisions about which variable to include in models were based on theory. In addition, we examined whether there was clustering by clinic but found no collinearity above -0.37 between clinic and any of the outcome or predictor variables.

We estimated sequential nested linear regression models to examine factors associated with three continuous dependent variables (SC information, SC motivation, and SC behavioral skills). Variables were selected based on theoretical relationships between constructs and drew from sexual and reproductive health/HIV and/or SC empirical/theoretical work in sub-Saharan African contexts and our prior work in Botswana. The independent variables were selected a priori to represent key demographic, relationship/partner, reproductive autonomy, fertility, violence, and stigma covariates. We examined mediation in all models. While we asked WLHIV about SC method use, this analysis focuses on the precursors of SC method use: actual SC method use was low (15% of women (n = 54) reported ever using a SC method, and 38 women had used SC methods within the past 5 years) and is not modeled here.

We estimated nested models for each dependent variable of interest (three models each for SC information, motivation, and behavioral skills, nine models total). Variables were added to all models to mirror a socio-ecological approach, first exploring intrapersonal variables, then interpersonal variables, and finally structural barriers. In the SC information models, we first looked at intrapersonal socio-demographic variables (including age, education level, relationship status, fertility desires, and SC motivation). Next, we added interpersonal variables (reproductive autonomy within relationships) because theoretically, reproductive autonomy might have an influence on SC information. In the final model, we added structural variables (internalized childbearing stigma and perceived community childbearing stigma) to the aforementioned variables. We examined the effect of these two types of stigma because theory suggested that internalized childbearing stigma and perceived community childbearing stigma might impact SC information.

In the SC motivation models, we first looked at intrapersonal socio-demographic variables (including age, education level, source of income, relationship status, number of living children) and SC information. Next, we added interpersonal variables (perceived partner willingness to use SC, and lifetime experiences of physical violence) to the aforementioned variables. Theoretically, perceived partner willingness to use SC might be associated with increased SC motivation, while experiences of violence may be associated with reductions in SC motivation. In the final model, we added structural-level stigma variables (anticipated stigma, internalized childbearing stigma, and perceived community childbearing stigma) to the previously listed variables. We added all three stigma variables in this model because theory suggested that all three types of stigma might be associated with SC motivation.

In the SC behavioral skills models, we first looked at a model containing intrapersonal socio-demographic variables (including recruitment site, age, education level, source of income, relationship status), fertility desires, SC information, and SC motivation. In the next model, we added the interpersonal variable perceived partner willingness to use SC to the aforementioned variables because theory suggested that perceived partner willingness might be associated with behavioral skills. In the final model, we added the structural internalized stigma variable because theoretically, internalized childbearing stigma was the stigma most likely to impact SC behavioral skills.

## Results

### Sample characteristics

Three hundred fifty-six (n = 356) WLHIV were enrolled in this study. Of 391 eligible WLHIV that were screened, 33 (8%) did not take part, most commonly citing time constraints. The mean age of participants was 33.6 years (SD=5.6, range 18-40 years) and over 80% reported secondary or higher level education (Table 1). Few women (11%) were married while most reported cohabiting (35%) or being in a relationship but not cohabiting (33%). Of those who reported having a current partner (n = 280), 90% (n = 251) said they knew the HIV status of their partner and 47% (n = 119) of those who said they knew their partner’s status said they were in sero-different relationships. Almost all women who had a current partner (96%) indicated they had disclosed that they were living with HIV to their partner. Women had been living with HIV for a mean of 8.5 years (SD=5.8, range 0-31 years, meaning that some participants had been diagnosed in the year of the study (2018) up to 31 years prior), almost all women (99%) were taking ART, but only 53% reported that a healthcare provider had told them they were currently virally suppressed. Almost 50% of the sample had been pregnant since being diagnosed with HIV and over half (57%) had ever used prevention of mother-to-child transmission (PMTCT) of HIV services. Lifetime experiences of IPV were reported by 31% of women (30% emotional, 19% physical, 12% sexual). Of women who had ever experienced violence, 33% had experienced violence within the last year.

**Table 1:** Characteristics of the sample of women living with HIV (n = 356)

Characteristic	Mean (SD) / Frequency (%)
**Demographics**	
Recruitment Location	
Hospital clinic	126 (35.4)
Peri-urban clinics	40 (11.2)
Urban clinics	190 (53.4)
Age - Mean	33.63 (5.6)
Education	
No formal education/ Pre-primary/ Primary	65 (18.3)
Secondary	222 (62.4)
Certificate/ Diploma/ Degree/ Post-grad	69 (19.4)
Main source of income	
Wage work	182 (51.9)
Casual work	37 (10.5)
Spouse/partner/family	22 (6.3)
Small business owner	71 (20.2)
Other/ Student/ Nothing	39 (11.1)
**Relationship Characteristics**	
Relationship status	
Married	38 (10.7)
Cohabiting, not married	124 (34.8)
In relationship, not cohabiting	117 (32.9)
No partner	76 (21.4)
Disclosed HIV status to current partner (n = 280)	268 (95.71)
Know HIV status of partner	
Yes	251 (89.6)
No	29 (10.4)
HIV-status of partner	
HIV-positive	132 (52.6)
HIV-negative	119 (47.4)
Have children with current partner	132/279 (47.3)
**Reproductive health and HIV care history**	
Total number of pregnancies	2.43 (1.4)
Number of years since HIV diagnosis	8.54 (5.8)
Currently taking ART	351 (98.6)
Told you are currently virally suppressed	185 (53.2)
Pregnancy since being diagnosed with HIV	169 (47.5)
Ever enrolled in PMTCT	178 (57.4)
**Fertility intentions**	
Desire for children/ more children in future	
Yes	243 (68.3)
No	113 (31.7)
**Lifetime experiences of intimate partner violence**	
Any form of lifetime violence	110 (30.90)
Emotional violence	108 (30.34)
Physical violence	69 (19.38)
Sexual violence	43 (12.08)
**Experiences of any intimate partner violence in the past 12 months (n = 110)**	36 (32.73)
**Reproductive autonomy scale** [includes decision-making, freedom from coercion, communication] (mean (SD); scale range)	39.02 (4.94) [12–45]
**SC information, motivation, behavioral skills**	
SC Information (mean (SD); scale range)	11.21 (2.7) [1–17]
SC Motivation (mean (SD); scale range)	11.17 (1.6) [3–12]
SC Behavioral skills (mean (SD); range)	3.00 (1.7) [0-6]
Self-efficacy for skills (mean (SD); range)	21.79 (2.9) [6–24]

### SC information

The overall sample mean for the scale (range of 0 to 17) was 11.01 (SD = 2.78), and the median was 11 (Table 2). Awareness of specific SC methods was generally low, except for male circumcision (83%). After male circumcision, the greatest proportion of participants were aware that viral suppression could be used by PLHIV to reduce the chance of transmitting HIV to a negative partner (64%). Awareness of vaginal insemination techniques, PrEP, timed condomless intercourse, and sperm washing were all markedly lower than awareness of male circumcision or viral suppression, with 40% or less of participants aware of these methods.

**Table 2:** Percentage of participants correctly answering each item of the safer conception method awareness scale (correct responses in bold)

Questions about SC in general and specific SC methods	True	False	Don’t know
1. It is possible for an HIV-positive woman to have an HIV-negative baby.	**93.5%**	2.5%	3.9%
2. HIV antiretroviral medications can reduce the risk of passing HIV to a baby.	**89.0%**	3.9%	7.0%
3. There are ways to make conception with an HIV-positive partner safer.	**85.4%**	2.8%	11.8%
4. There are ways to make conception with an HIV-negative partner safer.	**85.7%**	4.5%	9.8%
5. All options to make conception safer are very expensive.	10.4%	**75.0%**	14.6%
6. Waiting until one’s CD4 count is higher will reduce the risk of health complications to the mother during pregnancy.	**77.3%**	12.1%	10.7%
7. Having a sexually transmitted infection will increase the risk of passing HIV to an uninfected partner during unprotected sex.	**87.9%**	5.1%	7.0%
8. There are times during a woman’s cycle when she is most fertile (likely to become pregnant).	**73.9%**	5.1%	21.1%
9. Healthcare providers can offer advice to help make childbearing safer for women, their partners, and their children.	**90.7%**	4.2%	5.1%
10. If an HIV-positive person has an undetectable amount of HIV virus, it means that person is no longer able to infect someone else.	**30.6%**	51.7%	17.7%
11. Having the man ejaculate into condom/ container and manually inject semen into woman’s vagina is a way to reduce risk of HIV transmission if man is HIV-negative.	**40.2%**	16.9%	43.0%
12. Only having unprotected sex during the few days each month when the woman is most fertile will help to reduce the risk of HIV transmission to an uninfected partner.	**18.0%**	53.7%	28.4%
13. There is technology available that can cleanse a man’s sperm or semen of the HIV virus.	**10.7%**	32.6%	56.7%
14. Starting to take HIV medications early (as soon as diagnosed) helps reduce the risk of transmitting HIV to a sexual partner.	**61.5%**	25.8%	12.6%
15. HIV medications can be taken by an HIV-positive partner who wants to conceive with an HIV-negative partner in order to reduce the chance of transmitting HIV to the negative partner.	**64.3%**	18.5%	17.1%
16. HIV medications can be taken by an HIV-negative (or unknown status) partner that will reduce their risk of getting infected by their HIV-positive partner.	**34.3%**	46.6%	19.1%
17. An HIV-negative man can be circumcised as a way to reduce the chance of the man getting HIV during unprotected sex when a couple is trying to get pregnant.	**82.6%**	9.0%	8.4%
**Mean score (SD) for awareness of SC methods (scale range 0-17)**	**11.0 (2.8)**		
**Median score for awareness of SC methods**	**11**		

### Multivariate correlates of SC information, motivation, and behavioral skills

#### SC information models

In the first SC information model, after controlling for the effects of other variables, SC motivation was significantly associated with increased SC information (ß=0.20, p = 0.028). In the second SC information model, reproductive autonomy was significantly associated with increased SC information (ß=0.07, p = 0.029), while the relationship between SC motivation and SC information was mediated and was no longer significant. In the final SC information model, when we added stigma variables, the reproductive autonomy variable was no longer significantly associated with SC information, while both internalized childbearing stigma and perceived community childbearing stigma were negatively associated with SC information (ß=-0.50, p = 0.001 and ß=-0.09, p = 0.016, respectively). Variables in the model accounted for a significant amount of variability observed in information (*F*_(11,333)_ = 3.40, *p* <0.001, final model R-square value of 0.10) (Table 3).

**Table 3:** Multiple linear regression analysis of correlates of safer conception information

SC Information	Model 1			Model 2			Model 3		
VARIABLES	β	SE	95% CI	β	SE	95% CI	β	SE	95% CI
Desire more children in future	0.30	(0.34)	-0.37 - 0.97	0.31	(0.34)	-0.35 - 0.98	0.17	(0.33)	-0.48 - 0.83
SC motivation	0.20**	(0.09)	0.02 - 0.39	0.17*	(0.09)	-0.02 - 0.35	0.13	(0.10)	-0.06 - 0.32
Reproductive autonomy				0.07**	(0.03)	0.01 - 0.13	0.04	(0.03)	-0.02 - 0.11
Perceived community childbearing stigma							-0.09**	(0.04)	-0.80 - -0.21
Internalized childbearing stigma							-0.50***	(0.15)	-0.17 - -0.02
Observations	345			345			345		
R-squared	0.04			0.05			0.10		

Standard errors in parentheses

*** p < 0.01, ** p < 0.05, * p < 0.1

### SC motivation models

In the first SC motivation model, after controlling for the effects of other variables, SC information was significantly associated with increased motivation to use SC (ß=0.08, p = 0.013). In the second SC motivation model, when we added perceived partner willingness to use SC (ß=0.07, p = 0.008), and lifetime experiences of physical violence (ß=0.44, p = 0.050), they were associated with increased motivation to use SC but mediated the relationship between SC information and SC motivation, causing SC information to no longer be significant. In the final SC motivation model, the addition of stigma variables mediated the effect of some variables causing perceived partner willingness to use SC and lifetime experiences of physical violence to become non-significant. However, anticipated (ß=-0.06, p = 0.038) and internalized stigma (ß=-0.27, p = 0.002) were both associated with decreased SC motivation, while perceived community childbearing stigma was associated with increased SC motivation (ß=0.07, p = 0.003). Variables in the model accounted for a significant amount of variability observed in motivation (*F*_(17,321)_ = 5.07, *p* <0.001, final model R-square value of 0.21) (Table 4)

**Table 4:** Multiple linear regression analysis of correlates of safer conception motivation

SC motivation	Model 1			Model 2			Model 3		
VARIABLES	β	SE	95% CI	β	SE	95% CI	β	SE	95% CI
SC information	0.08**	(0.03)	0.02 - 0.14	0.06*	(0.03)	-0.00 - 0.12	0.04	(0.03)	-0.02 - 0.10
Perceived partner willingness to use SC				0.07***	(0.03)	0.02 - 0.12	0.03	(0.03)	-0.02 - 0.08
Lifetime experiences of physical violence				0.44**	(0.22)	0.00 - 0.89	0.39*	(0.22)	-0.03 - 0.82
Anticipated stigma							-0.06**	(0.03)	-0.12 - -0.00
Internalized childbearing stigma							-0.27***	(0.09)	-0.44 - -0.10
Perceived community childbearing stigma							0.07***	(0.02)	0.02 - 0.11
Observations	339			339			339		
R-squared	0.11			0.14			0.21		

Standard errors in parentheses

*** p < 0.01, ** p < 0.05, * p < 0.1

### SC behavioral skills models

In the first SC behavioral skills model, after controlling for the effects of other variables, SC information (ß=0.37, p < 0.001) and SC motivation (ß=0.64, p < 0.001) were both associated with increased SC behavioral skills. In the second model, when we added the perceived partner willingness to use SC variable, it was associated with increased SC behavioral skills (ß=0.53, p < 0.001), and SC information (ß=0.30, p < 0.001) and SC motivation (ß=0.48, p < 0.001) also remained associated with increased SC behavioral skills. However, the addition of the perceived partner willingness to use SC variable mediated the relationship between SC information, SC motivation, and SC behavioral skills. In the final SC behavioral skills model, the addition of internalized childbearing stigma was significant and was associated with decreased SC behavioral skills (ß=-0.80, p < 0.001). When the internalized stigma variable was added, SC information (ß=0.24, p < 0.001), motivation (ß=0.36, p = 0.001), and perceived partner willingness to use SC (ß=0.47, p < 0.001) all stayed positively associated with behavioral skills, but the relationships were mediated. Variables in the model accounted for a significant amount of variability observed in behavioral skills (*F*_(17,319)_ = 15.64, *p* <0.001, final model R-square value of 0.45) (Table 5)

**Table 5:** Multiple linear regression analysis of correlates of safer conception behavioral skills

SC Behavioral skills	Model 1			Model 2			Model 3		
VARIABLES	β	SE	95% CI	β	SE	95% CI	β	SE	95% CI
Desire more children in future	0.45	(0.45)	-0.43 - 1.33	0.69*	(0.39)	-0.08 - 1.46	0.53	(0.38)	-0.22 - 1.28
SC information	0.37***	(0.07)	0.23 - 0.51	0.30***	(0.06)	0.17 - 0.42	0.24***	(0.06)	0.12 - 0.36
SC motivation	0.64***	(0.12)	0.40 - 0.88	0.48***	(0.11)	0.27 - 0.70	0.36***	(0.11)	0.15 - 0.58
Perceived partner willingness to use SC				0.53***	(0.05)	0.42 - 0.64	0.47***	(0.05)	0.36 - 0.57
Internalized stigma							-0.80***	(0.17)	-1.12 - -0.47
Observations	337			337			337		
R-squared	0.24			0.41			0.45		

Standard errors in parentheses

*** p < 0.01, ** p < 0.05, * p < 0.1

## Discussion

In this study of WLHIV in Botswana, information about specific SC methods varied, with less than half being aware of vaginal insemination, PrEP, timed condomless intercourse, or sperm washing as SC methods. Furthermore, the data reveal that relationship and partner factors as well as various forms of stigma, including internalized childbearing stigma, anticipated stigma, and perceived community childbearing stigma, affect SC information, motivation, and behavioral skills. These findings suggest that if we do not address partner-level factors as well as stigma in SC interventions, we are missing key barriers to care. Although the IMB model served as the framework for understanding SC in this context, the discussion is organized to examine how IMB affects different types of factors. We discuss implications for intervention development throughout.

### SC information

While medical male circumcision and ART use for viral suppression were well-known SC methods, most other methods, including PrEP, were known by 40% of participants or less. Other studies in sub-Saharan Africa have similarly found low but varying information levels about SC methods [23, 25, 79]. While PrEP is a powerful SC option for sero-different couples, the low knowledge of PrEP is not surprising. Although the most recent Botswana HIV clinical care guidelines suggested that PrEP could be appropriate for sero-different couples attempting to conceive, at the time of the study, PrEP was not yet widely available and guidance on how to counsel couples on using this approach was limited [80]. Although rates of information were low for most SC methods, it is useful to know which factors were associated with that information. These findings are an important start to better understanding issues that need attention and how best to counsel and support WLHIV. In addition, the low method-specific information levels noted are concerning since almost half of the study participants reported they were in sero-different relationships and desired more children in the future. SC information is especially relevant for this group. However, 96% of participants said they had disclosed their status to their partner, and this bodes well for engaging their partners in reproductive healthcare.

In the era of U = U (Undetectable = Untransmittable or uninfectious), some might question why knowledge of various SC techniques matters since if viral suppression is achieved, the risk of HIV transmission to partners should be eliminated. However, viral suppression cannot be confirmed in all settings because routine viral load testing may not be available and simply providing ART does not ensure that all PLHIV in real world settings are virally suppressed and achieving the full benefits of treatment when trying to get pregnant [1, 18, 34, 81–83]. In a study in South Africa, 33% of WLHIV and 53% of men living with HIV (MLHIV) receiving ART who were attending a SC service for couples had detectable viral loads [18]. This is further supported by our own results, since only 53% of women in this study reported that a provider told them they were currently virally suppressed or undetectable. In addition, while 62-64% of women understood that being on ART can reduce their risk of transmitting HIV, our qualitative work suggests that women feel that no method is 100% effective at preventing the spread of HIV [34]. Our prior qualitative work in Botswana also found apathy about ART use among WLHIV, making SC approaches to compliment ART use even more relevant [34]. Similarly, a South African study among MLHIV who desired children found that men wanted to avoid using ART as treatment as prevention [79]. Clients would benefit from having information about a wide range of SC methods since one approach will not work for all couples and providing options supports reproductive choice [22, 66, 79]. Therefore, an intervention aimed at improving SC uptake would need to educate PLHIV about a range of SC techniques. Materials or posters that could be available in clinics would be helpful.

### Relationship and partner factors

While research shows that women should be offered SC services whether they arrive with their partner or not, the current study and other research highlight the importance of partner-level factors on SC motivation, behavioral skills, and ultimately, method uptake [18, 25, 84, 85]. Since many SC methods require full partner participation and these decisions happen between couples, there is a need to focus SC counseling at the couple-level and increase male engagement. However, identifying the right place to offer SC counseling to maximize uptake by couples is challenging. While some guidelines and research have suggested integrating SC counseling within family planning, antenatal, or post-natal care services, these services are often seen as female spheres, and few men attend [84, 86]. Leveraging existing services that target couples, such as integrating SC counseling and services within couples’ voluntary HIV counseling and testing or partner disclosure programs may help reach more couples with SC counseling and methods.

Another approach is to focus on male partner engagement as a way to increase SC uptake [87, 88]. Male partners have a strong impact on fertility and SC decisions [18, 24, 25, 31, 33] and interventions that acknowledge these dynamics are needed. Male partners likely need more information about SC methods, and identifying SC methods that men are comfortable with is critical to their use. While a study in South Africa with MLHIV who desired children found that men were enthusiastic for a clinic-based SC intervention [79], they also found structural barriers to participating in clinic-based interventions [87]. HIV-negative men in sero-different relationships may be reluctant to visit health centers for SC information and counseling. Community-based male engagement interventions may be more appealing and accessible to men. Counseling from community health workers in the home may help increase comfort and engagement. Also, providing SC information in community settings such as kgotla meetings (traditional community council meetings often headed by the village chief) may build public support and help reach more men. Interventions that work within men’s social networks have also been shown to improve uptake of HIV preventive services and may be especially relevant [89–91].

### The effects of stigma

WLHIV in Botswana face various forms of stigma that have negative but also unexpected positive associations for levels of SC information, motivation, and behavioral skills. Internalized childbearing stigma had a negative impact on SC information, motivation, and behavioral skills. Similarly, a Ugandan study found that internalized childbearing stigma was associated with lower odds of discussing childbearing intentions with providers, a key behavioral skill [92]. However, to our knowledge, the impact of internalized childbearing stigma on SC information and motivation has not previously been reported. In addition, anticipated HIV stigma from family members and healthcare providers had a negative impact on motivation to use SC. In our prior qualitative work in Botswana, we found that WLHIV anticipated stigma from healthcare providers and were afraid to discuss their fertility desires with them [38, 66]. Qualitative studies from Uganda and South Africa have similarly found that anticipated stigma is a barrier to seeking SC services [85, 93] and that MLHIV feel they cannot discuss SC with healthcare providers [79]. Given these qualitative findings, it is not surprising that that anticipated stigma had a negative impact on motivation to use SC.

While research has found that PLHIV perceive strong community disapproval associated with HIV and reproduction [30, 51, 53, 94, 95], perceived community childbearing stigma offered a more complicated picture. Perceived community childbearing stigma had a negative effect on SC information but had a positive effect on SC motivation. Although we tend to assume that stigma results in negative consequences, it seems plausible that if one perceives that there is community childbearing stigma, this might motivate one to use SC methods to reduce the chance of HIV infection to a partner and mitigate possible stigma. Supporting this idea, a study in Uganda found that greater perceived provider stigma of childbearing was associated with SC method use [19]. In addition, a Botswana study found that perceiving stigmatization from family and healthcare workers was associated with WLHIV not planning to have a child [96]. While in that case, perceiving stigma led to a desire not to have children, an alternate approach, as we saw in our study, is for stigma to motivate one to use a SC method that can allow one to have children more safely. Saleem and Pollard have suggested that having children can be a strategy that PLHIV use to conceal their status in order to avoid stigma and deal with the disruption that HIV poses to one’s reproductive identity [51]. Supporting this idea, a study from South Africa found that MLHIV see SC as an option to mitigate community-level HIV-stigma and that having children can restore their sense of value in society [87]. Using SC can therefore be seen as another coping strategy – a way to maintain one’s reproductive identity while reducing the chance of HIV transmission and the potential for stigma from one’s community.

The effects of stigma in this analysis were striking, and the need to address various forms of HIV stigma is a clear imperative of this research. When stigma variables were added to models, most relationships were mediated by the effects of stigma. These results show how important stigma is to SC information, motivation, and behavioral skills. Therefore, it is necessary to destigmatize childbearing for couples affected by HIV and address multiple forms of stigma. Normalizing discussions about pregnancy desires and SC for PLHIV and routinely assessing these needs at HIV care visits over time may help address anticipated stigma and signal to PLHIV that childbearing is a normal part of life that can be discussed within HIV care settings. Interventions for providers could focus on initiating fertility desire discussions, how to counsel women about a range of SC techniques, and values clarification related to reproduction among PLHIV to provide non-judgmental SRH services. While current Botswana HIV clinical guidelines offer healthcare providers limited guidance on how to support clients in need of SC methods [80], our prior qualitative research suggests that providers would like to know how to better support couples who want children and are receptive to receiving training [66]. We recommend policy changes in healthcare settings so that SC information dissemination becomes a routine part of obstetrics and gynecology care. In addition, it is important to destigmatize childbearing for PLHIV at the community-level. Botswana is a religious country, with 79% identifying as Christian [97]. Mass media campaigns that enlist church leaders to address how HIV stigma is not in accordance with their religious beliefs could facilitate the creation of supportive social spaces to challenge stigma, further community dialogue to reduce HIV stigma and contribute to the care and support of HIV-affected couples [98].

Interventions for PLHIV and couples are also needed. At the individual level, an intervention aimed at newly diagnosed PLHIV should be developed and could be offered before or as part of ART initiation in clinics. Such an intervention could focus on reproductive rights, the ability to have safe pregnancies, seeking clinical support, pre-conception wellness, optimizing treatment adherence, SC strategies, and the need for safe and effective family planning methods if pregnancy is not desired. This intervention could be offered within a healthcare setting or through community support groups. Training materials for providers and educational materials for clients that address many of these topics were developed in Kenya and could be adapted for Botswana [95, 99, 100]. We believe that such an intervention might help address internalized childbearing stigma and anticipated stigma. At the interpersonal level, a couples-based SC intervention for WLHIV and their sero-negative male partners that provides information about various SC methods, works on building specific behavioral skills to use SC methods, and tries to address internalized childbearing stigma and perceived community stigma by educating about reproductive rights should also be developed. To be most effective in Botswana, we recommend that such an intervention be based at the community-level.

### Strengths and Limitations

This study has strengths and limitations. This is the first quantitative study from Botswana to address SC, and the findings highlight areas that are important to address to improve SC uptake. The large sample size and the placement of study recruitment sites at public sector clinics suggest the results should be representative of WLHIV accessing care in the public sector. Many of the measures used were developed in sub-Saharan Africa, specifically to assess issues related to SC, which supports the applicability of the findings for this context. In addition, surveys were pretested in Setswana to ensure comprehension and local relevance. Also, local research assistants, who were not healthcare providers at the recruitment clinics, administered the surveys, thus limiting response and social desirability bias.

Despite these strengths, this study is not without limitations. The cross-sectional nature limits the ability to draw conclusions about causal relationships between variables. However, since this is the first survey of SC conducted in Botswana, the results are useful for intervention development. While most measures were developed in sub-Saharan Africa and known to be valid and reliable, we created some new measures. All new measures were evaluated through cognitive interviewing and were piloted to assess face and construct validity and reliability. However, it is possible that newly created SC behavioral skills scale did not capture all the relevant SC behavioral skills. In addition, while the IMB model guided the conceptual design of this study, we did not test the meditational relationships between the IMB constructs.

The study was conducted at urban and peri-urban sites, limiting the generalizability of the results to rural locations. While parts of Botswana are rural, the country is rapidly becoming more urban with 69% of the population residing in urban areas in 2018 [101], particularly in the south of the country, where this study took place.

This study was only conducted among WLHIV in HIV care. While the results are not generalizable to those not accessing care, few people in need of HIV care are not accessing it as 84% of those in need of treatment in Botswana are reported to be accessing ART through the national ART program [1]. It is possible that those who are accessing care have greater familiarity with SC methods: thus our results may present the best-case scenario in terms of SC information. However, since there are no formal SC services currently offered in Botswana in the public sector, levels of information, motivation, and behavioral skills to use SC methods are based on limited exposure to SC methods. In addition, while the most recent numbers from Botswana suggest 83-88% viral suppression in a trial setting [102], only 53% of women in this study reported that a provider had told them they were currently virally suppressed or undetectable. One of the clinics that we recruited from included a referral clinic for more complicated HIV cases (clients experiencing treatment failure) which may include a larger proportion of people who are non-adherent or do not achieve viral suppression for other reasons.

The study results are also not generalizable to men living with HIV. While we acknowledged that pregnancy decisions are made in couples and that male partners have a strong impact on fertility and SC decisions, we did not survey men in this study since far more women access HIV care and sexual and reproductive health services in this context. However, research on the feasibility, acceptability, and use of SC methods when the male partner is the HIV-affected partner is available from sub-Saharan African contexts [17, 18, 88]. Finally, although SC methods are particularly relevant for sero-different couples, partner HIV status was not part of this study’s eligibility criteria as both HIV sero-concordant and sero-different couples can benefit from SC services.

## Conclusions

These findings suggest that information, motivation, and behavioral skills will be important determinants of SC method use. Understanding the factors that affect these constructs can help target interventions so that deficits in information, motivation, and behavioral skills can be addressed. SC methods are an important HIV prevention strategy that can help couples reduce incident HIV cases during conception while also supporting the reproductive rights of PLHIV to achieve their desired family size. SC continues to be a relevant intervention because while viral suppression should address concerns about transmission, routine viral load testing is absent in many sub-Saharan contexts, and adherence is not always adequate to achieve suppression [1, 81, 82]. Therefore, along with ART, a range of SC techniques should be offered to HIV-affected couples as part of a comprehensive continuum of care.

## Supplementary Information

**Additional file 1.**

## Data Availability

The datasets used during the current study are available from the corresponding author on reasonable request.

## Abbreviations

ART: Antiretroviral therapy
CI: Confidence interval
DHMT: District health management team
HIV: Human immunodeficiency virus
IMB: Information, motivation, and behavioral skills
IPV: Intimate partner violence
MLHIV: Men living with HIV
PLHIV: People living with HIV
PMTCT: Prevention of mother-to-child transmission
PrEP: Pre-exposure prophylaxis
SC: Safer conception
SE: Standard error
SD: Standard deviation
WLHIV: Women living with HIV

## References

[1] UNAIDS. Botswana Country Factsheet 2019. 2019. http://www.unaids.org/en/regionscountries/countries/botswana. Accessed 31 May 2019.

[2] Mayondi GK, Wirth K, Morroni C, Moyo S, Ajibola G, Diseko M (2016). Unintended pregnancy, contraceptive use, and childbearing desires among HIV-infected and HIV-uninfected women in Botswana: a cross-sectional study. BMC Public Health.

[3] Government of Botswana. Botswana Second Generation HIV Antenatal Sentinel Serveillance Technical Report, 2011. Gaborone, Botswana; 2011.

[4] National AIDS Coordinating Agency. Botswana 2013 Global AIDS Response Report: Progress Report of the National Response to the 2011 Declaration of Commitments on HIV and AIDS. Gaborone; 2014.

[5] Doherty K, Arena K, Wynn A, Offorjebe OA, Moshashane N, Sickboy O (2018). Unintended Pregnancy in Gaborone, Botswana: A Cross-Sectional Study. Afr J Reprod Health.

[6] Gaolathe T, Wirth KE, Holme MP, Makhema J, Moyo S, Chakalisa U (2017). Botswana’s progress toward achieving the 2020 UNAIDS 90-90-90 antiretroviral therapy and virological suppression goals: A population-based survey. Lancet HIV.

[7] UNAIDS. UNAIDS Data 2018. Geneva; 2018. doi:10.15713/ins.mmj.3.

[8] Cohen MS, Chen YQ, McCauley M, Gamble T, Hosseinipour MC, Kumarasamy N (2011). Prevention of HIV-1 infection with early antiretroviral therapy. N Engl J Med.

[9] Donnell D, Baeten JM, Kiarie J, Thomas KK, Stevens W, Cohen CR (2010). Heterosexual HIV-1 transmission after initiation of antiretroviral therapy: a prospective cohort analysis. Lancet.

[10] Vernazza PL, Graf I, Sonnenberg-Schwan U, Geit M, Meurer A (2011). Preexposure prophylaxis and timed intercourse for HIV-discordant couples willing to conceive a child. AIDS.

[11] Baeten JM, Donnell D, Ndase P, Mugo NR, Campbell JD, Wangisi J (2012). Antiretroviral prophylaxis for HIV prevention in heterosexual men and women. N Engl J Med.

[12] Karim QA, Karim SSA, Frohlich JA, Grobler AC, Mansoor LE, Kharsany ABM (2010). Effectiveness and Safety of Tenofovir Gel, an antiretroviral microbicide, for the prevention of HIV Infection in women. Science (80-).

[13] Mmeje O, Cohen CR, Cohan D. Evaluating safer conception options for HIV-serodiscordant couples (HIV-infected female/HIV-uninfected male): A closer look at vaginal insemination. Infect Dis Obstet Gynecol. 2012;2012. 10.1155/2012/587651 PMC3423871 22927714

[14] Auvert B, Taljaard D, Lagarde E, Sobngwi-Tambekou J, Sitta R, Puren A (2005). Randomized, controlled intervention trial of male circumcision for reduction of HIV infection risk: The ANRS 1265 trial. PLoS Med.

[15] Gray RH, Kigozi G, Serwadda D, Makumbi F, Watya S, Nalugoda F (2007). Male circumcision for HIV prevention in men in Rakai, Uganda: a randomised trial. Lancet.

[16] Rodger AJ, Cambiano V, Bruun T, Vernazza P, Collins S, Lunzen J, Van (2016). Sexual Activity Without Condoms and Risk of HIV Transmission in Serodifferent Couples When the HIV-Positive Partner Is Using Suppressive Antiretroviral Therapy. JAMA..

[17] Mmeje O, van der Poel S, Workneh M, Njoroge B, Bukusi E, Cohen CR (2015). Achieving pregnancy safely: perspectives on timed vaginal insemination among HIV-serodiscordant couples and health-care providers in Kisumu, Kenya. AIDS Care.

[18] Schwartz SR, Bassett J, Holmes CB, Yende N, Phofa R, Sanne I (2017). Client uptake of safer conception strategies: implementation outcomes from the Sakh’umndeni Safer Conception Clinic in South Africa. J Int AIDS Soc.

[19] Wagner GJ, Linnemayr S, Goggin K, Mindry D, Sarah JB, Robinson FE (2017). Prevalence and Correlates of Use of Safer Conception Methods in a Prospective Cohort of Ugandan HIV-Affected Couples with Fertility Intentions. AIDS Behav.

[20] Heffron R, Ngure K, Velloza J, Kiptinness C, Quame-amalgo J, Oluch L (2019). Implementation of a comprehensive safer conception intervention for HIV-serodiscordant couples in Kenya: uptake, use and effectiveness. JIAS.

[21] Schwartz SR, Bassett J, Mutunga L, Yende N, Mudavanhu M, Phofa R (2019). HIV incidence, pregnancy, and implementation outcomes from the Sakh ’ umndeni safer conception project in South Africa : a prospective cohort study. Lancet HIV.

[22] Gutin SA, Harper GW, Amico KR, Bitsang C, Moshashane N, Harries J (2020). ‘I’m waiting for that’: Interest in the use of PrEP for safer conception in Botswana. Glob Public Health.

[23] Gwokyalya V, Beyeza-Kashesya J, Bwanika JB, Matovu JKB, Mugerwa S, Arinaitwe J (2019). Knowledge and correlates of use of safer conception methods among HIV-infected women attending HIV care in Uganda. Reprod Health.

[24] Ngure K, Baeten JM, Mugo N, Curran K, Vusha S, Heffron R (2014). My intention was a child but I was very afraid: fertility intentions and HIV risk perceptions among HIV-serodiscordant couples experiencing pregnancy in Kenya. AIDS Care.

[25] Wagner GJ, Woldetsadik MA, Beyeza-Kashesya J, Goggin K, Mindry D, Finocchario-Kessler S (2016). Multi-level Correlates of Safer Conception Methods Awareness and Attitudes among Ugandan HIV Clients with Fertility Intentions. Afr J Reprod Health.

[26] Kaida A, Kastner J, Ng C, Nyu NS-, Kusasira A, Kabakyenga J (2014). Barriers and Promoters to Uptake of Safer Conception Strategies among HIV-serodiscordant Couples with Fertility Intention in Mbarara, Uganda. AIDS Res Hum Retroviruses.

[27] Ngure K, Kimemia G, Dew K, Njuguna N, Mugo N, Celum C (2017). Delivering safer conception services to HIV serodiscordant couples in Kenya: perspectives from healthcare providers and HIV serodiscordant couples. J Int AIDS Soc.

[28] Schwartz SR, Bassett J, Sanne I, Phofa R, Yende N, Van Rie A (2014). Implementation of a safer conception service for HIV-affected couples in South Africa. AIDS.

[29] 10.1007/s10461-015-1026-x.

[30] Beyeza-Kashesya J, Ekstrom AM, Kaharuza F, Mirembe F, Neema S, Kulane A (2010). My partner wants a child: a cross-sectional study of the determinants of the desire for children among mutually disclosed sero-discordant couples receiving care in Uganda. BMC Public Health.

[31] Gutin SA, Namusoke F, Shade SB, Mirembe F (2014). Fertility Desires and Intentions among HIV-Positive Women during the Post-natal period in Uganda. Afr J Reprod Health.

[32] Bekker L-G, Black V, Myer L, Rees H, Cooper D, Mall S (2011). Guideline on safer conception in fertile HIV-infected individuals and couples. South Afr J HIV Med.

[33] Matthews LT, Crankshaw T, Giddy J, Kaida A, Smit Ja, Ware NC (2013). Reproductive decision-making and periconception practices among HIV-positive men and women attending HIV services in Durban, South Africa. AIDS Behav.

[34] Gutin SA, Harper GW, Moshashane N, Ramontshonyana K, Mompe A, Fleming PJ, et al. “I Did Not Know About All These”: Perceptions Regarding Safer Conception Methods by Women Living with HIV in Gaborone, Botswana. PLoS One. 2020;:1–18. doi:10.1371/journal.pone.0242992. 10.1371/journal.pone.0242992 PMC7707558 33259505

[35] 10.1080/09540121.2015.1093596.

[36] Matthews LT, Crankshaw T, Giddy J, Kaida A, Psaros C, Ware NC (2012). Reproductive counseling by clinic healthcare workers in Durban, South Africa: perspectives from HIV-infected men and women reporting serodiscordant partners. Infect Dis Obstet Gynecol.

[37] Saleem HT, Surkan PJ, Kerrigan D, Kennedy CE (2016). HIV Care Providers’ Communication with Patients About Safer Conception for People Living with HIV in Tanzania. Int Perspect Sex Reprod Health.

[38] Gutin SA, Harper GW, Bitsang C, Moshashane N, Harries J, Morroni C (2019). Perspectives about childbearing and pregnancy planning amongst people living with HIV in Gaborone, Botswana. Cult Health Sex.

[39] Rasmussen LM. Counselling clients to follow “the rules” of safe sex and ARV treatment. Culture, Health and Sexuality. 2013;15 SUPPL.4:537–52. doi:10.1080/13691058.2013.809606. 10.1080/13691058.2013.809606 23863077

[40] Ddumba-Nyanzi I, Kaawa-Mafigiri D, Johannessen H (2016). Barriers to communication between HIV care providers (HCPs) and women living with HIV about child bearing: A qualitative study. Patient Educ Couns.

[41] Colvin CJ, Konopka S, Chalker JC, Jonas E, Albertini J, Amzel A (2014). A Systematic Review of Health System Barriers and Enablers for Antiretroviral Therapy (ART) for HIV-Infected Pregnant and Postpartum Women. PLoS One.

[42] Goggin K, Mindry D, Beyeza-Kashesya J, Finocchario-Kessler S, Wanyenze R, Nabiryo C (2014). “Our Hands Are Tied Up”: Current State of Safer Conception Services Suggests the Need for an Integrated Care Model. Health Care Women Int.

[43] Kawale P, Mindry D, Phoya A, Jansen P, Hoffman RM (2015). Provider attitudes about childbearing and knowledge of safer conception at two HIV clinics in Malawi. Reprod Health.

[44] Matthews LT, Mukherjee JS (2009). Strategies for harm reduction among HIV-affected couples who want to conceive. AIDS Behav.

[45] Clouse K, Schwartz S, Van Rie A, Bassett J, Yende N, Pettifor A (2014). “What they wanted was to give birth; nothing else”: barriers to retention in option B+ HIV care among postpartum women in South Africa. J Acquir Immune Defic Syndr.

[46] Steiner RJ, Dariotis JK, Anderson JR, Finocchario-Kessler S (2013). Preconception care for people living with HIV: recommendations for advancing implementation. AIDS.

[47] Goggin K, Finocchario-Kessler S, Staggs V, Woldetsadik MA, Wanyenze RK, Beyeza-Kashesya J (2015). Attitudes, Knowledge, and Correlates of Self-Efficacy for the Provision of Safer Conception Counseling Among Ugandan HIV Providers. AIDS Patient Care STDS.

[48] Ong L, DeHaes J, Hoos A, Lammes F (1995). Doctor-Patient Communication: A Review of the Literature. Soc Sci Med.

[49] Keogh SC, Urassa M, Roura M, Kumogola Y, Kalongoji S, Kimaro D (2012). The impact of antenatal HIV diagnosis on postpartum childbearing desires in northern Tanzania: A mixed methods study. Reprod Health Matters.

[50] Evens E, Tolley E, Headley J, McCarraher DR, Hartmann M, Mtimkulu VT (2015). Identifying factors that influence pregnancy intentions: evidence from South Africa and Malawi. Cult Health Sex.

[51] Pollard R, Saleem H. Reproductive identities following an HIV diagnosis: Strategies in the face of biographical disruption. Cult Health Sex. 2019;:1–13. doi:10.1080/13691058.2019.1603399. 10.1080/13691058.2019.1603399 31012809

[52] Schaan MM, Taylor M, Gungqisa N, Marlink R (2016). Personal views about womanhood amongst women living with HIV in Botswana. Cult Health Sex.

[53] Kimemia G, Ngure K, Baeten JM, Celum C, Dew K, Njuguna N (2019). Perceptions of pregnancy occurring among HIV-serodiscordant couples in Kenya. Reprod Health.

[54] Kohler PK, Ondenge K, Mills La, Okanda J, Kinuthia J, Olilo G (2014). Shame, Guilt, and Stress: Community Perceptions of Barriers to Engaging in Prevention of Mother to Child Transmission (PMTCT) Programs in Western Kenya. AIDS Patient Care STDS..

[55] Fisher JD, Fisher WA, Peterson JL, DiClemente RJ (2000). Chapter 1. Theoretical Approaches to Individual-Level Change in HIV Risk Behavior. Handbook of HIV Prevention.

[56] Fisher JD, Fisher WA (1992). Changing AIDS-risk behavior. Psychol Bull.

[57] Smith LR, Amico KR, Shuper PA, Christie S, William A, Cornman DH (2013). Information, motivation, and behavioral skills for early pre-ART engagement in HIV care among patients entering clinical care in KwaZulu-Natal, South Africa. AIDS Care.

[58] Fisher JD, Fisher WA, Shuper PA, DiClemente RJ, Crosby RA, Kegler MC (2009). The Information-motivation-behavioral skills model of HIV preventive behavior. Emerging Theories in Health Promotion Practice and Research.

[59] Cornman DH, Kiene SM, Christie S, Fisher WA, Shuper PA, Pillay S (2008). Clinic-based intervention reduces unprotected sexual behavior among HIV-infected patients in KwaZulu-Natal, South Africa: results of a pilot study. J Acquir Immune Defic Syndr.

[60] Cornman DH, Christie S, Shepherd LM, MacDonald S, Amico KR, Smith LR (2011). Counsellor-delivered HIV risk reduction intervention addresses safer sex barriers of people living with HIV in KwaZulu-Natal, South Africa. Psychology & Health.

[61] Woldetsadik MA, Goggin K, Staggs VS, Wanyenze RK, Deborah JB (2016). Safer Conception Methods and Counseling: Psychometric Evaluation of New Measures of Attitudes and Beliefs Among HIV Clients and Providers. AIDS Behav.

[62] Crankshaw TL, Matthews LT, Giddy J, Kaida A, Ware NC, Smit JA (2012). A conceptual framework for understanding HIV risk behavior in the context of supporting fertility goals among HIV-serodiscordant couples. Reprod Health Matters.

[63] Upton RL, Dolan EM (2011). Sterility and Stigma in an Era of HIV / AIDS: Narratives of Risk Assessment among Men and Women in Botswana. Afr J Reprod Health.

[64] WHO. Women and Health: Today’s Evidence, Tomorrow’s Agenda. Geneva, Switzerland; 2009.

[65] Santelli JS, Lindberg LD, Orr MG, Finer LB, Speizer I (2009). Toward a multidimensional measure of pregnancy intentions: Evidence from the United States. Stud Fam Plann.

[66] Gutin SA, Harper GW, Moshashane N, Bitsang C, Harries J, Ramogola-Masire D (2020). What if they are pre-conception? What should we do?”: Knowledge, practices, and preferences for safer conception among women living with HIV and healthcare providers in Gaborone, Botswana. Front Glob Women’s Heal.

[67] Matthews LT, Burns BF, Bajunirwe F, Kabakyenga J, Bwana M, Ng C, et al. Beyond HIV-serodiscordance: Partnership communication dynamics that affect engagement in safer conception care. PLoS One. 2017;:1–17. doi:10.1371/journal.pone.0183131. 10.1371/journal.pone.0183131 PMC5589112 28880892

[68] Finocchario-Kessler S, Wanyenze R, Mindry D, Beyeza-Kashesya J, Goggin K, Nabiryo C (2014). “I May Not Say We Really Have a Method, It Is Gambling Work”: Knowledge and Acceptability of Safer Conception Methods Among Providers and HIV Clients in Uganda. Health Care Women Int.

[69] Kaida A, Lima VD, Andia I, Kabakyenga J, Mbabazi P, Emenyonu N, et al. The WHOMEN’s scale (women’s HAART optimism monitoring and EvaluatioN scale v.1) and the association with fertility intentions and sexual behaviours among HIV-positive women in Uganda. AIDS Behav. 2009;13. 10.1007/s10461-009-9553-y PMC3606958 19387819

[70] Schwartz SR, Rees H, Mehta S, Venter WDF, Taha TE, Black V (2012). High incidence of unplanned pregnancy after antiretroviral therapy initiation: findings from a prospective cohort study in South Africa. PLoS One.

[71] Uganda Bureau of Statistics. Demographic and Health Survey 2011. 2012;:1–461.

[72] Upadhyay UD, Dworkin SL, Weitz TA, Foster DG (2014). Development and Validation of a Reproductive Autonomy Scale. Stud Fam Plann.

[73] Loll D, Fleming PJ, Manu A, Morhe E, Stephenson R, King EJ (2020). Reproductive autonomy and pregnancy decision-making among young Ghanaian women. Glob Public Health.

[74] Loll D, Fleming PJ, Stephenson R, King EJ, Morhe E, Manu A (2021). Factors associated with reproductive autonomy in Ghana. Cult Health Sex.

[75] Garcia-Moreno C, Jansen HAFM, Heise L, Watts C. WHO Multi-country Study on Women’s Health and Domestic Violence against Women: Initial results on prevalence, health outcomes and women’s responses. Geneva; 2005.

[76] Earnshaw VA, Smith LR, Chaudoir SR, Amico KR, Copenhaver MM (2013). HIV stigma mechanisms and well-being among PLWH: A test of the HIV Stigma Framework. AIDS Behav.

[77] Sileo KM, Wanyenze RK, Kizito W, Reed E, Brodine SK, Chemusto H (2019). Multi-level Determinants of Clinic Attendance and Antiretroviral Treatment Adherence Among Fishermen Living with HIV/AIDS in Communities on Lake Victoria, Uganda. AIDS Behav.

[78] Kwena Z, Kimbo L, Darbes LA, Hatcher AM, Helova A, Owino G (2021). Testing strategies for couple engagement in prevention of mother-to-child transmission of HIV and family health in Kenya: study protocol for a randomized controlled trial. Trials..

[79] Matthews LT, Greener L, Khidir H, Psaros C, Harrison A, Mosery FN (2021). “It really proves to us that we are still valuable”: Qualitative research to inform a safer conception intervention for men living with HIV in South Africa. PLoS One.

[80] Botswana MOH, Masa. Handbook of the Botswana Integrated HIV Clinical Care Guidelines. Gaborone, Botswana; 2016.

[81] Mills EJ, Nachega JB, Bangsberg DR, Singh S, Rachlis B, Wu P (2006). Adherence to HAART: A systematic review of developed and developing nation patient-reported barriers and facilitators. PLoS Medicine.

[82] Keiser O, Chi BH, Gsponer T, Boulle A, Orrell C, Phiri S (2011). Outcomes of antiretroviral treatment in programmes with and without routine viral load monitoring in southern Africa. AIDS.

[83] Davies NECG, Ashford G, Bekker L-G, Chandiwana N, Cooper D, Dyer SJ (2018). Guidelines to support HIV-affected individuals and couples to achieve pregnancy safely: Update 2018. South Afr J HIV Med.

[84] Patwa MC, Bassett J, Holmes L, Mutunga L, Mudavanhu M, Makhomboti T (2019). Integrating safer conception services into primary care: providers ’ perspectives. BMC Public Health.

[85] Crankshaw TL, Mindry D, Munthree C, Letsoalo T, Maharaj P (2014). Challenges with couples, serodiscordance and HIV disclosure: Healthcare provider perspectives on delivering safer conception services for HIV-affected couples, South Africa. J Int AIDS Soc.

[86] Mindry D, Wanyenze RK, Beyeza-Kashesya J, Woldetsadik MA, Finocchario-Kessler S, Goggin K (2017). Safer Conception for Couples Affected by HIV: Structural and Cultural Considerations in the Delivery of Safer Conception Care in Uganda. AIDS Behav.

[87] Mathenjwa M, Khidir H, Milford C, Mosery N, Rambally Greener L, Pratt MC (2021). Acceptability of an Intervention to Promote Viral Suppression and Serostatus Disclosure for Men Living with HIV in South Africa: Qualitative Findings. AIDS Behav.

[88] Khidir H, Psaros C, Greener L, Neil KO, Mathenjwa M, Lizzie FNM (2018). Developing a Safer Conception Intervention for Men Living with HIV in South Africa. AIDS Behav.

[89] Ajilore O (2015). Identifying peer effects using spatial analysis: the role of peers on risky sexual behavior. Rev Econ Househ.

[90] Neblett RC, Davey-Rothwell M, Chander G, Latkin CA (2011). Social network characteristics and hiv sexual risk behavior among urban African American women. J Urban Heal.

[91] Grieb SM, Davey-Rothwell M, Latkin CA (2012). Social and sexual network characteristics and concurrent sexual partnerships among urban African American high-risk women with main sex partners. AIDS Behav.

[92] Beyeza-Kashesya J, Wanyenze RK, Goggin K, Finocchario-Kessler S, Woldetsadik MA, Mindry D, et al. Stigma gets in my way: Factors affecting client-provider communication regarding childbearing among people living with HIV in. PLoS One. 2018;:1–14. doi:10.1371/journal.pone.0192902. 10.1371/journal.pone.0192902 PMC5819785 29462171

[93] Matthews LT, Bajunirwe F, Kastner J, Sanyu N, Akatukwasa C, Ng C, et al. “I Always Worry about What Might Happen Ahead”: Implementing Safer Conception Services in the Current Environment of Reproductive Counseling for HIV-Affected Men and Women in Uganda. Biomed Res Int. 2016;2016:1–9. http://search.ebscohost.com/login.aspx?direct=true&site=eds-live&db=asx&AN=113562725. 10.1155/2016/4195762 PMC4802028 27051664

[94] Beyeza-Kashesya J, Kaharuza F, Mirembe F, Neema S, Ekstrom AM, Kulane A (2009). The dilemma of safe sex and having children: challenges facing HIV sero-discordant couples in Uganda. Afr Health Sci.

[95] Mmeje O, Njoroge B, Akama E, Leddy A, Breitnauer B, Darbes L (2016). Perspectives of healthcare providers and HIV-affected individuals and couples during the development of a Safer Conception Counseling Toolkit in Kenya: stigma, fears, and recommendations for the delivery of services. AIDS Care.

[96] Schaan MM, Taylor M, Marlink R (2014). Reproductive behaviour among women on antiretroviral therapy in Botswana: mismatched pregnancy plans and contraceptive use. African J AIDS Res.

[97] Central Intelligence Agency. Botswana. The World Fact Book. 2021.

[98] Campbell C, Skovdal M, Gibbs A (2011). Creating social spaces to tackle AIDS-related stigma: Reviewing the role of church groups in sub-saharan Africa. AIDS Behav.

[99] Brown J, Njoroge B, Akama E, Breitnauer B, Leddy A, Darbes L (2016). A novel safer conception counseling toolkit for the prevention of HIV: A mixed-methods evaluation in Kisumu, Kenya. AIDS Educ Prev.

[100] Mmeje O, Njoroge B, Akama E, Leddy A, Breitnauer B, Darbes L, et al. Safer Conception Toolkit for HIV-affected Individuals and Couples and Healthcare Providers. HIVE Online. 2016. https://hiveonline.org/safer-conception-toolkit-for-hiv-affected-individuals-and-couples-and-healthcare-providers/. Accessed 21 Apr 2021. 10.1080/09540121.2016.1153592 PMC4942319 26960581

[101] United Nations Department of Economic and Social Affairs/ Population Division. World Urbanization Prospects, the 2018 revision: Botswana. World Urbanization Prospects: The 2018 Revision. 2018. https://esa.un.org/unpd/wup/Country-Profiles/. Accessed 17 Jun 2017.

[102] Makhema J, Wirth KE, Holme MP, Gaolathe T, Mmalane M, Kadima E (2019). Universal Testing, Expanded Treatment, and Incidence of HIV Infection in Botswana. N Engl J Med.

