# Supplementary material for: Relationship, partner factors and stigma are associated with safer conception information, motivation, and behavioral skills among women living with HIV in Botswana
Source: BMC Public Health. 2021 Dec 8;21:2231. doi: 10.1186/s12889-021-12268-5 (PMC8653588; doi:10.1186/s12889-021-12268-5)
Supplement: Supplementary file 1 — Additional file 1. [file 12889_2021_12268_MOESM1_ESM.docx]

**Appendix 2. Behavioral skills to use safer conception scale**

| **Behavioural skills to use SC** | | |
| --- | --- | --- |
| **Qs. #** | **Item** | **Responses** |
|  | A. Have you ever had a discussion with a healthcare provider about preventing pregnancy? | 1= Yes  0= No |
|  | B. How certain are you that you could start discussions with a healthcare provider about preventing pregnancy? | 1= I cannot do this at all  2= I probably cannot do it  3= I probably can do it  4= I can definitely do this |
|  | A. Have you ever had a discussion with a healthcare provider about wanting to become pregnant? | 1= Yes  0= No |
|  | B. How certain are you that you could start discussions with a healthcare provider about wanting to become pregnant? | 1= I cannot do this at all  2= I probably cannot do it  3= I probably can do it  4= I can definitely do this |
|  | A. Have you ever had a discussion with a healthcare provider about how to make conception safer if you want to become pregnant in the future? | 1= Yes  0= No |
|  | B. How certain are you that you could start discussions with a healthcare provider about ways to make conception safer if you wanted to become pregnant in the future? | 1= I cannot do this at all  2= I probably cannot do it  3= I probably can do it  4= I can definitely do this |
|  | A. Have you and your current partner ever talked to each other about wanting to have children together? | 1= Yes  0= No |
|  | B. How certain are you that you could start discussions with your partner about having children together? | 1= I cannot do this at all  2= I probably cannot do it  3= I probably can do it  4= I can definitely do this |
|  | A. Have you and your current partner ever talked about ways to make conception safer if you wanted to become pregnant in the future? | 1= Yes  0= No |
|  | B. How certain are you that you could start discussions with your partner about ways to make conception safer if you wanted to become pregnant in the future? | 1= I cannot do this at all  2= I probably cannot do it  3= I probably can do it  4= I can definitely do this |
|  | A. Have you ever taken steps to improve your health before becoming pregnant? | 1= Yes  0= No |
|  | B. How certain are you that you could take steps to improve your health before becoming pregnant? | 1= I cannot do this at all  2= I probably cannot do it  3= I probably can do it  4= I can definitely do this |
